# Data-independent acquisition mass spectrometry in severe rheumatic heart disease (RHD) identifies a proteomic signature showing ongoing inflammation and effectively classifying RHD cases

**DOI:** 10.1186/s12014-022-09345-1

**Published:** 2022-03-22

**Authors:** M. Taariq Salie, Jing Yang, Carlos R. Ramírez Medina, Liesl J. Zühlke, Chishala Chishala, Mpiko Ntsekhe, Bernard Gitura, Stephen Ogendo, Emmy Okello, Peter Lwabi, John Musuku, Agnes Mtaja, Christopher Hugo-Hamman, Ahmed El-Sayed, Albertino Damasceno, Ana Mocumbi, Fidelia Bode-Thomas, Christopher Yilgwan, Ganiyu A. Amusa, Esin Nkereuwem, Gasnat Shaboodien, Rachael Da Silva, Dave Chi Hoo Lee, Simon Frain, Nophar Geifman, Anthony D. Whetton, Bernard Keavney, Mark E. Engel, the RHDGen Network Consortium

**Affiliations:** 1 AFROStrep Research Group, Department of Medicine, University of Cape Town, Cape Town, South Africa; 2 Division of Cardiovascular Sciences, School of Medical Sciences, Faculty of Biology, Medicine and Health, The University of Manchester, Manchester, UK; 3 Manchester Heart Institute, Manchester University NHS Foundation Trust, Manchester, UK; 4 Stoller Biomarker Discovery Institute, Faculty of Biology, Medicine and Health, University of Manchester, Manchester, UK; 5 Division of Paediatric Cardiology, Department of Paediatrics and Child Health, Red Cross War Memorial Children’s Hospital and University of Cape Town, Cape Town, South Africa; 6 Division of Cardiology, University of Cape Town & Groote Schuur Hospital, Cape Town, South Africa; 7 Cardiology Department of Medicine, Kenyatta National Hospital, University of Nairobi, Nairobi, Kenya; 8 Departments of Adult and Pediatric Cardiology, Uganda Heart Institute, Kampala, Uganda; 9 University Teaching Hospital–Children’s Hospital, University of Zambia, Lusaka, Zambia; 10 Rheumatic Heart Disease Clinic, Windhoek Central Hospital, Windhoek, Namibia; 11 Department of Cardiothoracic Surgery, Alshaab Teaching Hospital, Alazhari Health Research Center, Alzaiem Alazhari University, Khartoum, Sudan; 12 Faculty of Medicine, Eduardo Mondlane University/Nucleo de Investigaçao, Departamento de Medicina, Hospital Central de Maputo, Maputo, Mozambique; 13 Faculdade de Medicina, Universidade Eduardo Mondlane, Maputo, Mozambique; 14 Division of Non Communicable Diseases, Instituto Nacional de Saude, Vila de Marracuene, Mozambique; 15 Departments of Paediatrics, Jos University Teaching Hospital, Jos, Plateau State Nigeria; 16 Department of Medicine and Cape Heart Institute (CHI), University of Cape Town, Cape Town, South Africa; 17 Division of Informatics, Imaging, and Data Sciences, University of Manchester, Manchester , UK; 18 Department of Surgery, University of Nairobi, Nairobi, Kenya; 19 Department of Medicine, University of Jos and Jos University Teaching Hospital, Jos, Nigeria; 20 School of Health Sciences, Faculty of Health and Medical Sciences, University of Surrey, Guildford, UK; 21 Faculty of Biosciences and Medicine, University of Surrey, Guildford, UK

**Keywords:** Rheumatic heart disease, Biomarker, Inflammatory response, Adiponectin, Complement component C7, Fibulin-1

## Abstract

**Background:**

Rheumatic heart disease (RHD) remains a major source of morbidity and mortality in developing countries. A deeper insight into the pathogenetic mechanisms underlying RHD could provide opportunities for drug repurposing, guide recommendations for secondary penicillin prophylaxis, and/or inform development of near-patient diagnostics.

**Methods:**

We performed quantitative proteomics using Sequential Windowed Acquisition of All Theoretical Fragment Ion Mass Spectrometry (SWATH-MS) to screen protein expression in 215 African patients with severe RHD, and 230 controls. We applied a machine learning (ML) approach to feature selection among the 366 proteins quantifiable in at least 40% of samples, using the Boruta wrapper algorithm. The case–control differences and contribution to Area Under the Receiver Operating Curve (AUC) for each of the 56 proteins identified by the Boruta algorithm were calculated by Logistic Regression adjusted for age, sex and BMI. Biological pathways and functions enriched for proteins were identified using ClueGo pathway analyses.

**Results:**

Adiponectin, complement component C7 and fibulin-1, a component of heart valve matrix, were significantly higher in cases when compared with controls. Ficolin-3, a protein with calcium-independent lectin activity that activates the complement pathway, was lower in cases than controls. The top six biomarkers from the Boruta analyses conferred an AUC of 0.90 indicating excellent discriminatory capacity between RHD cases and controls.

**Conclusions:**

These results support the presence of an ongoing inflammatory response in RHD, at a time when severe valve disease has developed, and distant from previous episodes of acute rheumatic fever. This biomarker signature could have potential utility in recognizing different degrees of ongoing inflammation in RHD patients, which may, in turn, be related to prognostic severity.

**Supplementary Information:**

The online version contains supplementary material available at 10.1186/s12014-022-09345-1.

## Introduction

The morbidity and mortality of rheumatic heart disease (RHD) is chiefly due to damage to the cardiac valves, consequent on an autoimmune reaction to Group A Streptococcal infection (typically, childhood sore throat). RHD is the only cardiovascular disease of global impact that has been shown to be completely preventable [1]. Poor social conditions, overcrowding, and limited access to medical resources are key enablers of RHD, which remains a major source of morbidity and mortality, in low and middle-income countries (LMICs) [2]. In excess of 40 million people are currently living with RHD worldwide [3]; most are in countries where advanced medical technologies such as percutaneous or surgical intervention are not accessible [4]. The Global Burden of Disease study has shown that RHD affects nearly five million more people than HIV and causes about 10 million disability adjusted life years lost globally.

Group A Streptococcus (GAS) is the etiological agent triggering Acute Rheumatic Fever (ARF), with evidence of molecular mimicry by the M protein on the bacteria, which shares an α-helical coiled structure with cardiac proteins such as myosin [5]. Antibodies to the M protein cross-react with heart tissues, leading to carditis and other systemic manifestations such as arthritis [6, 7]. The current dominant (but yet to be proved) understanding, is that progression to chronic RHD occurs through a pathway that includes repeated episodes of subclinical ARF in genetically susceptible individuals and interactions between host genes, GAS infections and social conditions of poverty [8].

RHD demonstrates a wide spectrum of symptoms and signs, with no single available confirmatory laboratory test; this adds to the difficulty in the diagnosis and treatment of early RHD cases [9]. Current diagnostic measures for ARF rely on the 2015 revised Jones criteria [10] incorporating echocardiography images of the heart valves [11]; however, the availability of echocardiography is highly limited in poorer countries. A striking mismatch between high prevalences of RHD and low prevalences of previously diagnosed ARF in developing countries has been observed [12, 13] indicating that a significant proportion of ARF cases are undetected, or undetectable with current tools, and there is a missed opportunity to identity and intervene in, those at risk for progression to severe RHD [10, 14]. Given the human and financial cost of this inability to recognize the disease until late in its course, a better understanding of the biological underpinnings of ARF and subsequent progression may present important targets for prevention and treatment. This study sought to complement our recent GWAS study confirming an association between RHD and genetic susceptibility loci in African individuals [15] through the identification of a plasma protein signature of RHD that may aid biological understanding of the processes involved, and potentially point towards economically feasible interventions to prevent severe RHD in poorer countries based upon repurposing of readily available and inexpensive medicines.

Mass spectrometry of clinical specimens using the SWATH-MS technique implements a Data-Independent Acquisition (DIA) approach for precision identification and accurate quantification of proteins [16]. Briefly, the approach begins with the generation of precursor fragments coupled with further sequentially fragmented windows across the entire mass to charge ratio range. These mass spectra chromatograms are compared to a spectral library with a spectral scoring strategy employed as an in-silico, label-free protein quantification method. SWATH-MS data have been successfully subjected to various informatics techniques, including machine learning (ML) algorithms, to identify and characterize the differentially expressed proteins from the resultant digitized SWATH maps [17]. Here we identify candidate protein biomarkers for ARF and RHD, by applying ML methodology to proteomic data acquired using SWATH-MS, in severe cases of RHD and controls recruited from peri-urban settings across Africa.

## Materials and methods

### Study design

Two-hundred and fifteen patients with severe RHD, and 230 healthy controls, of various ethnicities recruited in peri-urban settings across the African continent, were included in this study. A breakdown of the contributing countries and sites is shown in Additional file 1: Table S1. There was no age restriction of the cases, and the controls were ethnically matched individuals with no echocardiographic evidence of RHD and who were older than 15 years of age. Case severity was determined by an experienced clinician, who assessed each heart valve lesion referring to echocardiographic images, and categorised valve disease for severity according to the Gewitz/ACC criteria [10]. Informed consent was provided by each participant before inclusion into the study. After the consent process, 5 ml of blood were obtained through standard procedures by a trained on-site nurse and transported for processing to the Cardiovascular Genetics laboratory at the University of Cape Town. Briefly, blood tubes were centrifuged at 3000 rpm for 10 min and plasma aliquoted into vials for storage at −80 °C. The plasma samples of cases and controls were then subjected to SWATH-MS at the Stoller Biomarker Discovery Centre, University of Manchester.

### SWATH-MS proteomics

Samples were quality-checked, assigned a unique ID and cases and controls were randomized and prepared for mass spectrometry by tryptic hydrolysis after immunoaffinity depletion of the 12 major proteins found in plasma. To counteract batch effects following machine cleaning, we repeatedly tested plasma from pooled samples or a commercial standard until the Total Ion Current (TIC) Chromatogram stabilised, before running patient samples. Digitized proteomic maps were generated through SWATH-MS analysis performed on a 6600 TripleTOF mass spectrometer (Sciex, Warrington, UK) coupled to a Dionex Ultimate 3000 HPLC (Dionex, Thermo, UK), with specific mass spectrometric conditions (including isolation window size and overlap and total cycle time) as previously described [18].

Spectral libraries were generated by TransProteomic Pipeline (version 4.8.0) [19]. X!Tandem (version 2015.04.01.1) [20] was used to interrogate the SWATH-MS files generated from the samples. More specifically, the samples were pooled together to create a final set of 12 fractions and processed, generating 12 files that were searched against the appropriate database with X!Tandem. These files were further processed with the TransProteomic Pipeline, containing *xinteract*, *InterPropherParser* and *spectrast*, to generate the spectral library. SWATH maps were generated by OpenMS (version 2.0.1) [21] and MSproteomicstools (version 0.4.3). pyProphet (version 0.18.3) was used for the False Discovery Rate (FDR) calculations of the resulting transition groups. Feature alignment tools were used to align multiple pyProphet files with the corrected retention times and FDR scores. As the aligned SWATH maps contain transition-level information, MSstats() function from the R package MSstats [22] (version 3.13.5) was used to infer protein-level quantification. Parameters chosen were “top3” option for parameter “featureSubset” and normalisation with Tukey-Median Polish (TMP). Coefficient of variance (CV) analysis between technical injection replicates was performed on the resulting MSstats-processed data, with samples allowed to go forward to downstream analysis if the median and 75% quantiles were 20% and 30% maximum, respectively. Proteins present in at least 40% of the samples were retained in the following biomarker analysis [23]. The 12 purposely physically immunodepleted proteins were removed in silico prior to statistical analysis*.*

### Statistical analysis

Proteomic data was log_2_ transformed to stabilize the variance and reduce heteroscedasticity. Baseline phenotypic characteristics were compared between case and control groups using Mann–Whitney U tests for continuous variables and Chi-squared tests for proportions. As some cases were taking Warfarin, we removed proteins known to be Vitamin-K dependent. Relationships between medications prevalent among cases (chiefly Warfarin and penicillin) and individual proteins were explored using Student’s t-tests. Pearson correlation coefficients of protein expression with BMI and age were calculated among case and control samples, and we tested for interaction between case/control status and sex in expression of each protein. An unadjusted bivariate comparison of all proteins between cases and controls was carried out using Student’s t-tests applied to log2 proteomics data; p-values from this analysis were corrected for multiple comparisons using the Bonferroni method.

Feature selection was undertaken using the Boruta algorithm [24], which implements a random forest (RF) procedure comparing each candidate feature’s performance in a classification model with respect to that of a randomly created ‘shadow’ feature. Boruta has wide application in feature selection [25, 26] and has recently been applied to SWATH-MS data [27]. Boruta has been shown to be effective in permutation based feature selection [28]. The Boruta algorithm also has the merit of incorporating data from all collinearly associated proteins instead of randomly selecting one among them, as some other algorithms do. Log2 transformed proteomics data were randomly split into training and testing sets in a ratio of 7:3. The Boruta R package (version 7.0.0), was deployed with the parameter *ntree*, which defines the number of trees to grow, set to 500 and the parameter *maxRuns*, which specifies maximum runs the algorithm will iterate, set to 4000; these settings were chosen through an initial training of the model on a subset of the data.

In order to test the robustness of biomarkers detected by Boruta algorithm, the LASSO (Least Absolute Shrinkage and Selection Operator) logistic regression method was applied to the same training and testing datasets as used for Boruta algorithm. glmnet() function from R package glmnet (version 4.1–2) was used to carry out LASSO regression.

The glm() function in R was used to implement a logistic regression (LR) model to yield adjusted betas and per-marker AUCs for each log2 scaled proteomic feature that had emerged as significant from the Boruta analysis. BMI, age and sex were included in the model, as was a BMI*age interaction term. Twenty-three patients with missing BMI information (including 8 controls and 15 cases) were removed from these analyses. The cumulative AUC for the addition of each biomarker, in order of its Boruta importance, was calculated using the Cstat() function from the DescTools package.

Enrichment testing using the list of proteins identified by the Boruta algorithm was performed using ClueGo (version 2.5.7), a plug-in application in Cytoscape (version 3.8.2). The following databases were used: GO Biological Process; GO Molecular Functions; GO Immune System Process; KEGG; Reactome Pathways; Wiki Pathways. Following the approach used by others in similar analyses of plasma samples [29], we used the SWATH plasma reference library of 2,559 proteins as background in our principal analyses (analyses using the whole genome as background are presented in Additional file 1: Data). Only pathways with p-value < 0.05 (calculated using a two-sided hypergeometric test and Bonferroni step down correction) and a minimum of two proteins per pathway were considered.

## Results

### Demographic information

Among 445 participants in the study, there were 215 cases of severe RHD and 230 controls. Demographic baseline data are shown in Table 1. RHD is typically a disease of young people and as age-matching was not carried out in population collection, we found cases were significantly younger than controls (p = 0.014; Table 1). Sixty-four RHD patients were below the age of 18 years, whereas only 13 controls were below the age of 18 years old. Also, RHD cases had lower BMI than controls (p = 6.02e−12; Table 1). We therefore explored relationships between age, BMI and protein levels in the cohort prior to the case–control proteomic analyses. BMI and age in the cases were correlated with Pearson correlation coefficient r = 0.63, compared to r = 0.23 in control samples; the higher correlation in cases is mainly due to the presence of participants younger than 18 in the case cohort (Additional file 1: Fig. S1). Subsequent LR analyses were therefore adjusted for age, sex, BMI and age*BMI interaction. Regarding medication differences between cases and controls, 111 cases and zero controls in the study were receiving secondary prophylaxis for RHD, comprising regular benzathine penicillin G injections. Twenty-three cases and zero controls were identified as anticoagulated with warfarin. One case received both penicillin G injections and warfarin. Neither penicillin nor warfarin treatment (after the removal of proteins known to be affected by warfarin) was a significant factor in explaining protein differences between cases and controls.

**Table 1:** Baseline characteristics of included study participants. Data presented as median (IQR) or percentange (%). P-values obtained using the Mann-Whitney U test.

Characteristics	Cases (n = 215)^*^	Controls (n = 230)^*^	p-value
Age (years)	28 (16–41)	29 (23–41)	0.014
Gender, Male n (%)	63 (29.3%)	75 (32.6%)	0.45
BMI (kg/m^2^)	20.6 (16.3–24.7)	23.8 (20.8–29.2)	6.02e-12
BMI in participants ≥ 18yrs	23.0 (19.8–25.9)	23.9 (20.9–29.6)	0.005
NYHA Index	I = 4; II = 150 III = 36; IV = 10	N/A	N/A
Number of participants taking penicillin prophylaxis	111	0	N/A
Number of participants taking anticoagulation	23	0	N/A

^*^15 cases and 8 controls have BMI data missing. Missing data are removed from the statistical computation

### Proteomic baseline data

A total of 940 proteins were quantified in the blood samples and 366 proteins, present in at least 40% of the samples, were kept for downstream analysis (Additional file 1: Fig. S2). The principal reason for protein dropout was abundance level, rather than unacceptable levels of variation. Among these 366 proteins, no significant differences of protein expression were observed between participants taking warfarin or penicillin, compared to those not taking medication (pairwise t-test, adjusted p-value = 1). Correlation coefficients of protein expression and BMI or age were in general weak, and not systematically different between cases and controls (Additional file 1: Fig. S3). No protein showed significantly different expression in males than in females, and there were no significant interactions between case/control status and sex in protein expression (Additional file 1: Fig. S4).

### Boruta machine learning analyses

**Fig. 1:**
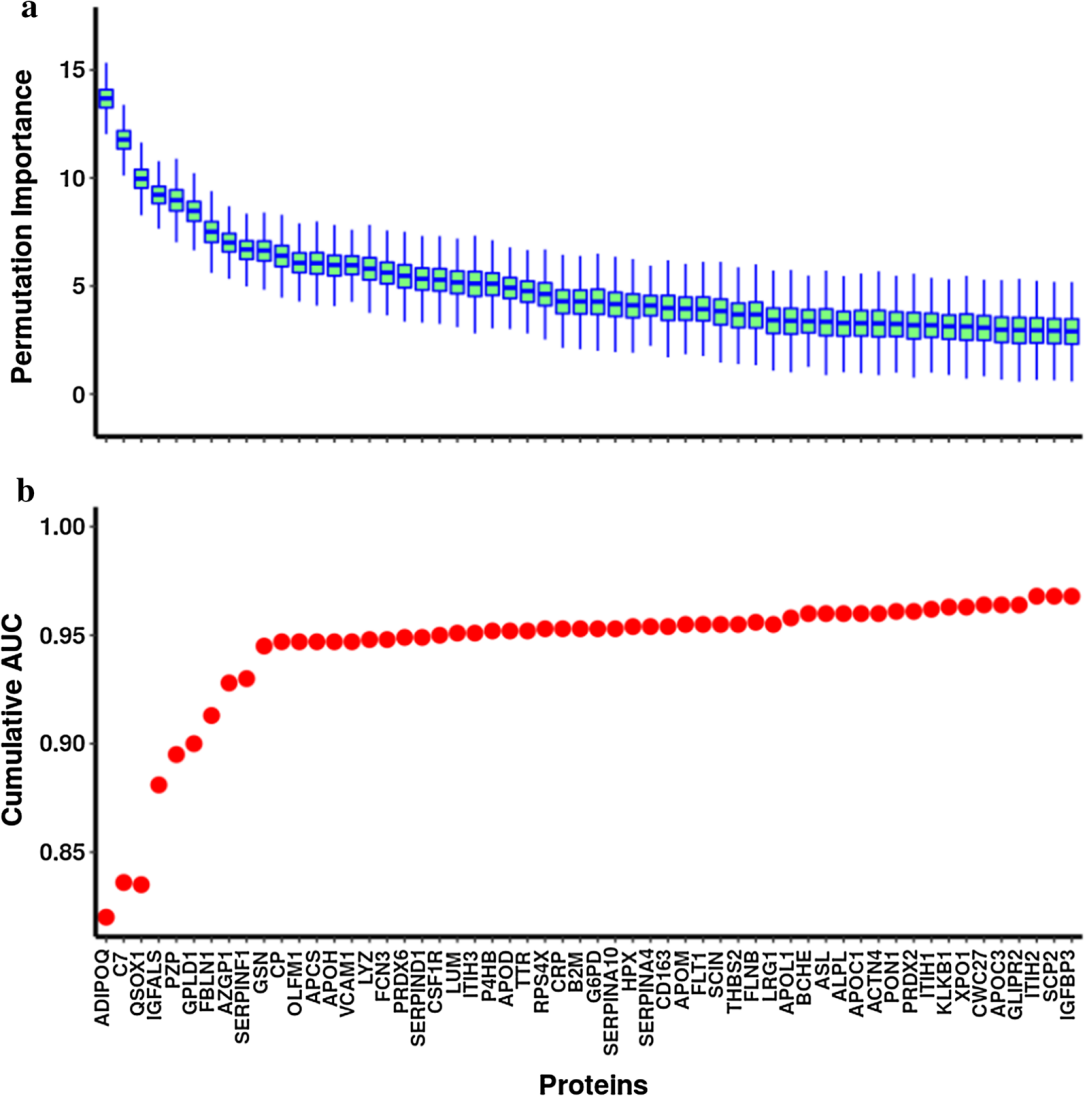
**a** Boxplot representing the permutation importance of the 56 proteins (from 215 cases; 230 controls) found to be significant by the Boruta algorithm. UniProt IDs are presented in Table 2. **b** Cumulative AUC for Boruta-identified biomarkers calculated from logistic regression analysis

Fold change analyses showed a total of 84 proteins that exhibited significant differences between cases and controls with adjusted p-values < 0.05 (Additional file 1: Table S2). Using the Boruta algorithm, 56 features were identified as important; these are presented in order of their Boruta importance in Table 2. Figure 1a shows the boxplots of the permutation importance of the 56 proteins in order with an emphasis on the top six proteins. Adiponectin (Q15848) and complement factor C7 (P10643) are the strongest differentially expressed proteins in this analysis, followed by quiescin sulfhydryl oxidase 1 (O00391), insulin-like growth factor binding protein acid labile subunit (P35858), pregnancy zone protein (P20742) and glycosylphosphatidylinositol specific phospholipase D1 (P80108). Twenty-four of the proteins identified by the Boruta algorithm were also identified by LASSO regression (Additional file 1: Table S3). However, the Boruta algorithm identified some important additional biomarkers, for example, quiescin sulfhydryl oxidase 1 (O00391), a known marker of cardiac disease, that LASSO regression did not detect.

**Table 2:** List of biomarkers identified from Boruta package with their log2-scaled mean expression in cases, controls, log2 fold change, mean permutation importance (meanImp); and with Odds Ratios (ORs), 95% Confidence Interval (CI), p-values and AUCs from single-marker LR models adjusted for age, sex, BMI, and age*BMI

UniProt ID	ProteinName	Mean of log2-scaled expression in cases	Mean of log2-scaled expression in controls	Log2-fold change	meanImp	OR with 95% CI	P value	AUC
Q15848	ADIPOQ	11.21	10.08	1.14	13.67	1.18 [1.13–1.24]	2.00e−12	0.820
P10643	C7	16.72	16.00	0.72	11.76	3.40 [2.41–4.93]	2.14e−11	0.815
O00391	QSOX1	12.49	11.96	0.52	9.95	1.27 [1.12–1.47]	5.58e−04	0.774
P35858	IGFALS	15.40	15.99	− 0.60	9.20	0.34 [0.23–0.48]	2.16e−09	0.799
P20742	PZP	16.64	15.79	0.85	8.95	2.25 [1.73–3.00]	7.98e−09	0.794
P80108	GPLD1	12.63	13.30	− 0.67	8.45	0.40 [0.29–0.54]	2.94e−09	0.799
P23142	FBLN1	13.86	13.31	0.55	7.49	1.96 [1.46–2.68]	1.44e−05	0.792
P25311	AZGP1	16.57	17.07	-0.49	7.00	0.35 [0.24–0.49]	5.30e−09	0.794
P36955	SERPINF1	15.19	15.66	-0.47	6.66	0.33 [0.22–0.49]	6.07e−08	0.785
P06396	GSN	16.92	17.49	− 0.57	6.63	0.39 [0.28–0.54]	2.86e−08	0.787
P00450	CP	20.34	19.91	0.44	6.39	2.34 [1.67–3.35]	1.49e−06	0.781
Q99784	OLFM1	10.93	10.53	0.40	6.06	1.09 [1.05–1.14]	5.16e−05	0.770
P02743	APCS	16.32	16.92	− 0.60	6.04	0.36 [0.26–0.50]	1.19e−09	0.795
P02749	APOH	18.54	18.98	− 0.44	5.94	0.38 [0.26–0.54]	2.28e−07	0.784
P19320	VCAM1	10.77	10.19	0.58	5.95	1.09 [1.05–1.14]	4.17e−05	0.773
P61626	LYZ	12.45	12.00	0.46	5.78	1.09 [1.05–1.15]	1.50e−04	0.771
O75636	FCN3	14.02	14.82	− 0.81	5.60	0.60 [0.47–0.76]	2.65e−05	0.811
P30041	PRDX6	14.82	14.44	0.37	5.44	1.41 [1.14–1.99]	1.93e−02	0.770
P05546	SERPIND1	18.07	18.52	− 0.45	5.31	0.40 [0.29–0.55]	5.20e−08	0.787
P07333	CSF1R	11.08	10.59	0.49	5.26	1.08 [1.03–1.13]	2.99e−03	0.761
P51884	LUM	17.03	16.78	0.25	5.15	1.52 [1.12–2.08]	7.48e−03	0.757
Q06033	ITIH3	15.51	15.16	0.35	5.08	1.73 [1.27–2.39]	6.97e−04	0.768
P07237	P4HB	16.41	15.69	0.72	5.08	1.02 [0.99–1.05]	1.63e−01	0.743
P05090	APOD	17.00	17.48	− 0.48	4.90	0.49 [0.36–0.66]	2.84e−06	0.778
P02766	TTR	17.61	18.21	− 0.60	4.73	0.49 [0.37–0.64]	1.73e−07	0.784
P62701	RPS4X	12.94	13.36	− 0.41	4.61	0.96 [0.90–1.02]	1.53e−01	0.747
P02741	CRP	13.94	12.77	1.17	4.27	1.13 [1.08–1.19]	1.25e−06	0.793
P61769	B2M	12.01	11.53	0.48	4.25	1.06 [1.01–1.11]	1.17e−02	0.761
P11413	G6PD	18.30	17.83	0.47	4.25	1.01 [0.97–1.07]	5.83e−01	0.743
Q9UK55	SERPINA10	12.92	13.13	-0.22	4.13	0.63 [0.42–0.90]	2.60e−02	0.769
P02790	HPX	21.43	21.87	-0.43	4.07	0.31 [0.20–0.45]	1.30e−08	0.793
P29622	SERPINA4	15.16	15.55	− 0.39	4.08	0.44 [0.30–0.62]	4.65e−06	0.771
Q86VB7	CD163	11.00	10.66	0.34	3.97	1.06 [1.02–1.11]	8.43e−03	0.753
O95445	APOM	16.09	16.54	− 0.45	3.92	0.93 [0.85–1.00]	4.80e−02	0.751
P17948	FLT1	13.21	13.51	− 0.30	3.91	0.99 [0.96–1.02]	6.26e−01	0.743
Q9Y6U3	SCIN	15.26	15.83	− 0.57	3.80	0.35 [0.25–0.48]	3.02e−10	0.800
P35442	THBS2	12.24	11.83	0.42	3.63	1.06 [1.02–1.10]	2.83e−03	0.760
O75369	FLNB	18.95	18.23	0.72	3.64	1.03 [1.01–1.06]	2.12e−02	0.756
P02750	LRG1	17.10	16.72	0.39	3.39	1.63 [1.26–2.12]	2.72e−04	0.768
O14791	APOL1	13.31	13.73	− 0.42	3.36	0.46 [0.34–0.62]	4.13e−07	0.788
P06276	BCHE	13.58	14.07	− 0.48	3.35	0.50 [0.36–0.70]	5.44e−05	0.789
P04424	ASL	16.30	15.70	0.60	3.31	0.99 [0.97–1.02]	5.62e−01	0.743
P05186	ALPL	13.55	13.83	− 0.27	3.25	0.94 [0.91–0.98]	1.74e−03	0.757
P02654	APOC1	15.45	15.95	− 0.50	3.26	0.59 [0.46–0.76]	4.60e−05	0.774
O43707	ACTN4	18.53	18.14	0.39	3.24	1.07 [1.03–1.11]	7.18e−04	0.760
P27169	PON1	16.08	16.63	− 0.55	3.22	0.54 [0.40–0.72]	4.19e−05	0.790
P32119	PRDX2	12.60	12.75	− 0.14	3.14	0.95 [0.90–1.00]	3.03e−02	0.750
P19827	ITIH1	18.66	19.01	− 0.34	3.16	0.45 [0.31–0.64]	1.49e−05	0.767
P03952	KLKB1	15.75	16.10	− 0.35	3.07	0.43 [0.30–0.62]	7.24e−06	0.782
O14980	XPO1	15.74	15.31	0.43	3.09	1.05 [1.00–1.11]	6.13e−02	0.753
Q6UX04	CWC27	14.37	14.74	− 0.38	3.04	0.96 [0.94–0.99]	1.44e−02	0.752
P02656	APOC3	15.32	15.93	− 0.61	2.96	0.71 [0.58–0.86]	5.84e−04	0.764
Q9H4G4	GLIPR2	12.70	12.28	0.42	2.93	1.01 [0.97–1.05]	7.13e−01	0.743
P19823	ITIH2	19.26	19.54	− 0.28	2.92	0.54 [0.37–0.76]	4.83e−04	0.760
P22307	SCP2	14.59	14.09	0.50	2.89	1.04 [1.00–1.07]	3.36e−02	0.752
P17936	IGFBP3	13.75	14.11	− 0.36	2.88	0.53 [0.37–0.73]	1.94e−04	0.763

### Logistic regression

Results of the marker-by-marker logistic regression analyses adjusted for age, sex, BMI and age*BMI, for each of the 56 proteins identified by the Boruta algorithm, are presented in Table 2. The top marker from the Boruta analyses, Adiponectin, was higher in cases than controls, exhibiting an OR for disease per unit increase on the log2 scale (ie per doubling) of 1.18 [95% CI 1.13–1.24]; p = 2.00e−12. The second placed marker by the Boruta algorithm, complement component C7, had the highest absolute case–control difference of any biomarker in the LR model, with OR = 3.40 [95% CI 2.41–4.93]; p = 2.14e−11. Among other significant markers, Fibulin-1, a known component of cardiac valve matrix, was higher in cases than controls, potentially indicating ongoing significant valve damage in these chronic RHD patients (OR = 1.96; [95% CI 1.46–2.68]; p = 1.44e−05). Also, in keeping with previous analyses [30], we found the complement-activating protein Ficolin-3 to be lower in cases than controls (OR = 0.60; [95% CI 0.47–0.76]; p = 2.65e−05). Ficolin-3 had a strong classification ability similar to Adiponectin and C7 with an individual AUC of 0.81. The cumulative AUC from the logistic regression analyses is shown in Fig. 1b. Incorporating the top 6 biomarkers in the model yielded an AUC of over 90% and incorporating the top 12 biomarkers yielded an AUC of ~ 0.95 (Table 2). Thus, the use of SWATH-MS based discovery proteomics identified a candidate biomarker signature that accurately discriminates RHD patients from controls.

### Pathway enrichment

**Fig. 2:**
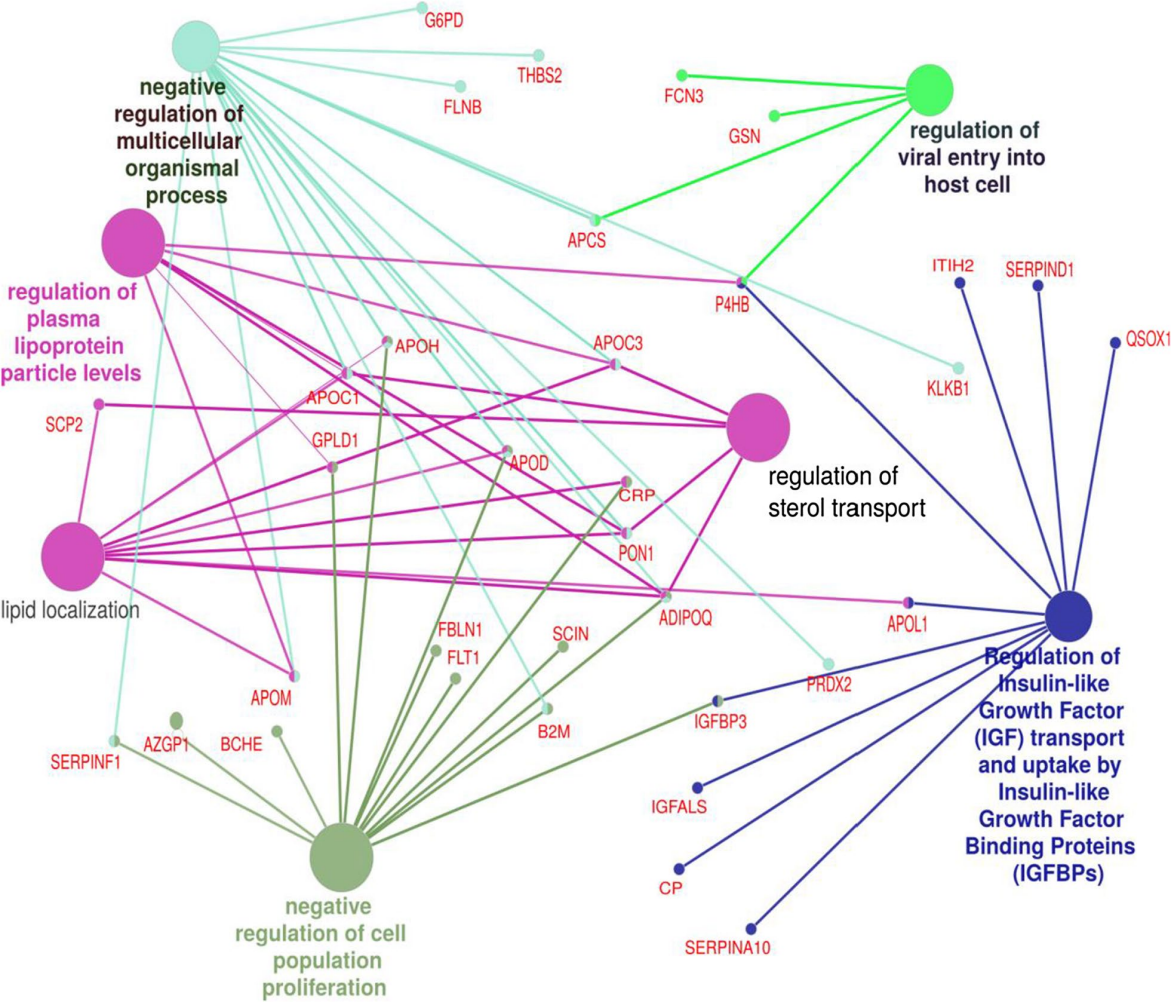
Functionally grouped networks of enriched pathways from ClueGO. For the full enrichment analysis results see Additional file 1: Table S4

Statistically significantly enriched pathways identified by ClueGo functional enrichment conducted on the Boruta-identified proteins are presented in Additional file 1: Table S4. A functionally grouped network of pathways is shown in Fig. 2. Enriched pathways confirmed our inference from the individual protein analyses that the activity of protein networks involved in inflammatory mechanisms were significantly different between cases and controls. For example, proteins involved in the Insulin like Growth Factor (IGF) and IGF-binding protein (IGFBP) pathways were significantly enriched (FDR-adjusted p = 1.70e−04) which are of known importance in autoimmunity [31]. Pathways of previously unsuspected relevance in RHD included serine-type endopeptidase inhibitors (FDR-adjusted p = 4.94e−05), including members of the Serpin family involved in stabilization of the extracellular matrix and inhibiting clotting proteins; and lipoprotein metabolism (FDR-adjusted p = 1.30e−04). Subsidiary analyses using the whole genome as background produced results highly congruent with the plasma reference library analyses (Additional file 1: Table S5).

## Discussion

In this study of geographically and ethnically diverse African patients with severe RHD and healthy controls, we identified a proteomic signature consistent with ongoing inflammation, during what has typically been considered a “burned out” phase of disease—when severe chronic valve disease is established.

Previous plasma proteomic studies of RHD have involved smaller numbers of patients than the present study: Mukherjee et al. [32] studied six patients with rheumatic mitral stenosis and six controls; Gao et al. [33] studied 40 RHD patients and 40 controls; and Wu et al. [34] carried out the only previous study of comparable size to the present investigation, involving 160 RHD patients and 160 healthy controls. There was minimal overlap between the proteins identified in those studies and the present investigation, which is the first to employ a machine learning approach to identify differentially expressed proteins. Proteomic studies of rheumatic human valves replaced at surgery offer the potential to more directly interrogate pathological processes, however these have involved only small numbers of patients, due to limited availability of specimens for study (recently reviewed by Lumngwena et al. [35]). Moreover, while such studies of valve tissue provide directly pathologically relevant information, they do not necessarily inform the basis for a potential field diagnostic. In the following, we discuss certain of the proteins that showed most significant differences between cases and controls and their potential relevance to RHD.

Adiponectin was the top protein identified in the Boruta and logistic regression analyses. Plasma adiponectin was a mean of 2.2 fold higher in cases than controls. Adiponectin has a complex relationship to inflammation, being currently thought to act as either an anti-inflammatory or a pro-inflammatory protein dependent on context [36]. In the context of diabetes, obesity and coronary artery disease, adiponectin is lower in cases than controls and inversely correlated with C-reactive protein (CRP) levels. By contrast, levels are higher in cases of rheumatoid arthritis, Systemic Lupus Erythematosus (SLE) and inflammatory bowel disease than controls. Thus elevation of adiponectin appears to be a specific autoimmune marker in the context of inflammation, in keeping with the disease process in RHD.

Complement factor 7 was the second most important protein in the Boruta and logistic regression analyses. Plasma C7 was a mean 1.6 fold higher in cases than controls. Unlike some other complement components, C7 is not considered an acute phase reactant, and it is the only terminal complement component not predominantly synthesised by hepatocytes [37]. C7 is often the limiting factor for terminal complement complex generation, and has been found at higher levels in plasma of diabetic patients with kidney disease [38]. Thus far there is no evidence for plasma C7 levels being altered in rheumatoid or autoimmune diseases. The combination of Adiponectin and C7 elevation in cases compared with controls together is therefore, to the best of our knowledge, unique to RHD among inflammatory diseases studied so far, and suggests their combination could have diagnostic utility.

Quiescin sulfhydryl oxidase 1 (QSOX1) was the third most important protein in the machine learning analyses. QSOX1 was on average 43% higher in cases than in controls. When fully adjusted for age/sex/BMI/age*BMI in logistic regression analyses, it fell to 29th position among the identified proteins, but remained statistically significantly different between cases and controls (OR = 1.27 [95% CI 1.12–1.47]; p = 5.58e−04). QSOX1 catalyses disulphide bond formation in fibroblasts, and supports ECM assembly in fibroblast cultures. It has been described as a marker of acute heart failure [39] and is higher in patients admitted with MI who later go on to develop LV dysfunction [40], in which situation it is thought to originate from the infarct border zone. QSOX1 has not previously been implicated in rheumatic or other heart valve disease.

Fibulin-1 is an extracellular matrix protein strongly expressed during development in the cardiac cushions, from which the heart valves develop, and in adult valve tissue [41, 42]. Plasma fibulin-1 levels have been suggested to be an early plasma marker of aortic stenosis [43]. Levels have been positively associated with N-terminal pro-BNP, and left atrial size [44], and fibulin is hypothesised to play a key role in determining aortic stiffness [45]. Our data showing a 46% higher mean value plasma fibulin-1 in RHD cases compared to controls, particularly when coupled with the pro-inflammatory signature constituted by other proteins, tends to support the notion of ongoing valve damage in late-stage RHD. However, this observation could also be consistent with left atrial size increase consequent upon mitral stenosis or regurgitation among a proportion of the cases.

We found Ficolin-3 levels to be about 43% lower in RHD cases than controls. Ficolin-3 is one of three ficolin proteins that bind to microbial surface residues, and play key roles together with the Mannose-binding lectin (MBL)-associated serine proteases 1 and 2 in the cleavage of complement components 4 and 2 to form the C3 convertase C4b2a [46]. The lectin pathway, of which Ficolin-3 is the most abundant plasma component, has been implicated in RHD by multiple previous studies; Ficolin 3 itself binds to the highly conserved N-acetyl-beta-D-glucosamine (GlcNAc) antigen, the main carbohydrate antigen of the Group A Streptococcus cell wall. Recently, a focused ELISA based study of serum Ficolin-3 concentrations showed a 30% lower serum ficolin-3 among 179 patients with a history of rheumatic fever compared to 170 healthy controls, a result strongly in concordance with our large-hypothesis experiment [30]; although a smaller recent study of Egyptian adolescents did not confirm this result [47]. It is possible that either consumption of Ficolin-3 by an ongoing inflammatory process, or a genetic predisposition to lower Ficolin-3 levels resulting in a greater propensity for streptococcal sore throat to progress to acute rheumatic fever among cases, may explain the association we and others have shown between severe RHD and lower plasma Ficolin-3. Further research will be required to distinguish these possibilities.

Taken together, our results strongly suggest an ongoing inflammatory process involving damage to the cardiac valves among these cases of severe RHD, which to date has remained an unresolved question. Of note, over 50% of the case population were treated with secondary penicillin prophylaxis, and we observed no difference in proteomic profile among those cases who were, and who were not, taking penicillin prophylaxis. This suggests that recent undiagnosed episodes of rheumatic fever would be an unlikely explanation for our observations. This is important in light of alternative plausible hypotheses for the drivers of progressive valve severity that are emerging. For example there is previous work showing that myocarditis remained in its active phase in patients with ARF, months after the disease ventured into the quiescent phase [48] suggesting that continuous valve damage may occur in a similar fashion in chronic RHD patients, with evidence of a continuum of inflammation due to the presence of high levels of CRP [49]. Elsewhere Karthikeyan and colleagues have suggested that a major driver of persistent inflammation and progression of valve disease may be related to the hemodynamic burden and turbulence created by transvalvular pressure gradients across damaged valves [50]. Of interest, Rifaie et al. reported that high concentrations of inflammatory markers present in the sera of patients with chronic rheumatic valvular heart disease subsequently disappeared after administration of anti-inflammatory drugs [51]. Clinical observation tends to support the notion of ongoing valve damage distant from ARF episodes—for example, while pure mitral regurgitation dominates in the young, mixed valvular pathology is the most common finding in chronic RHD, indicating progression [52]. Our results suggest these clinical changes reflect ongoing inflammation-driven valvular scarring and remodelling occurring in RHD, even distant from recurrent episodes of ARF.

Our analyses were able to distinguish a six-protein signature of severe RHD (ADIPOQ, C7, QSOX1, IGFALS, PZP, GPLD1) that correctly classified over 90% of cases; incorporation of the top 12 proteins enabled correct classification of over 95% of cases. Certain features of the signature appear, from the literature, to confer specificity—the combination of high Adiponectin and high C7, higher levels of Fibulin-1, and lower levels of Ficolin-3 in cases. If ongoing inflammation were shown to have prognostic importance in chronic RHD, the protein signature could be used to attempt to stratify RHD patients, and potentially identify opportunities for drug repurposing in future studies. A similar protein signature identifying ARF would be of even greater utility in low-resource settings, where access to experts trained in clinical cardiovascular evaluation, and the use of echocardiography, is very limited. Similar studies to ours will be necessary in ARF patients and controls to investigate this question.

This study has limitations. Although it is the largest study thus far, the only one to date to incorporate machine learning, and the first to use the SWATH-MS or proteomics methodology, replication of our findings in a second cohort of similar size would be of value. Incorporation of genetic information could enable a “Mendelian randomisation” approach to distinguish causal from non-causal association—this could be of particular value, for example, in the case of Ficolin-3 where lower levels could be due to either genetic predisposition or enhanced consumption by an ongoing inflammatory process. Such experiments would require larger samples. Adiponectin exists in three isoforms (trimer, hexamer and multimer) which are known to have differential properties in, for example, induction of chemokine expression in vitro [53]*.* Our approach could not distinguish these different isoforms, which would require alternative analytic platforms. It is therefore possible that we have underestimated the importance of a particular isoform of Adiponectin. Some of the proteins we identified as among the strongest biomarkers do not, as yet, have plausible mechanisms linking them to RHD; further research will be required to discover these.

In summary, we have identified a plasma protein signature of rheumatic heart disease that suggests an ongoing inflammatory process in the chronic phase of the condition. A small number of proteins considered together accurately classified chronic, severe RHD cases distinct from healthy controls. This work may could contribute to opportunities for drug repurposing, guide recommendations for prophylaxis, and/or inform development of near-patient diagnostics.

## Supplementary Information

**Additional file 1:** **Figure S1.** Scatter plots of BMI and age in cases and controls with their density plots in each group. **Figure S2.** Percentage of missing data in proteins with dashed red line representing the cutoff line. **Figure S3.** Pearson correlation coefficients of protein expression with BMI/Age among case and control samples. Correlation coefficients are in general weak, and not systematically different between cases and controls. **Figure S4.** Errorbar plots of protein expressions in females (x-axis) and males (y-axis) in case and control samples, respectively. The dashed lines are identity lines. **Table S1.** The eight African countries contributing participants in the study. **Table S2.** Mean of log2-scaled protein expression quantities in cases and controls, log2 foldchange between cases and controls, p-values from student t-test and adjusted p-values for multiple comparisons (only proteins with adjusted p value<0.05 are shown here). **Table S3.** Comparisons of protein signatures identified by Boruta algorithm and LASSO regression. **Table S4.** Pathways from ClueGO analysis of input proteins identified by Bortua algorithm by using plasma library reference (n = 2559). **Table S5.** Pathways from ClueGO analysis of input proteins identified by Bortua algorithm by using whole human genome reference. List of RHDGen Network Consortium Members

## Data Availability

Scripts and processed data accompanying the paper are available on the github repository: https://github.com/jyangUK/Rheumatic_heart_disease. The mass spectrometry proteomics data have been deposited to the ProteomeXchange Consortium via the PRIDE [56] partner repository with the dataset identifier PXD030598. Data access will be limited to the purpose of Rheumatic Heart Disease research, per ethical permissions for our study, and regulated by a Data Access Committee (Chair: ME). Further details on data accessibility are available from the Corresponding Author.
