# Supplementary material for: Data-independent acquisition mass spectrometry in severe rheumatic heart disease (RHD) identifies a proteomic signature showing ongoing inflammation and effectively classifying RHD cases
Source: Clin Proteomics. 2022 Mar 22;19:7. doi: 10.1186/s12014-022-09345-1 (PMC8939134; doi:10.1186/s12014-022-09345-1)
Supplement: Supplementary file 1 — Additional file 1: Figure S1. Scatter plots of BMI and age in cases and controls with their density plots in each group. Figure S2. Percentage of missing data in proteins with dashed red line representing the cutoff line. Figure S3. Pearson correlation coefficients of protein expression with BMI/Age among case and control samples. Correlation coefficients are in general weak, and not systematically different between cases and controls. Figure S4. Errorbar plots of protein expressions in females (x-axis) and males (y-axis) in case and control samples, respectively. The dashed lines are identity lines. Table S1. The eight African countries contributing participants in the study. Table S2. Mean of log2-scaled protein expression quantities in cases and controls, log2 foldchange between cases and controls, p-values from student t-test and adjusted p-values for multiple comparisons (only proteins with adjusted p value<0.05 are shown here). Table S3. Comparisons of protein signatures identified by Boruta algorithm and LASSO regression. Table S4. Pathways from ClueGO analysis of input proteins identified by Bortua algorithm by using plasma library reference (n = 2559). Table S5. Pathways from ClueGO analysis of input proteins identified by Bortua algorithm by using whole human genome reference. List of RHDGen Network Consortium Members [file 12014_2022_9345_MOESM1_ESM.pdf]

Supplementary file for the paper "Data independent acquisition mass spectrometry in severe Rheumatic Heart Disease (RHD) identifies a proteomic signature showing ongoing inflammation and effectively classifying RHD cases"

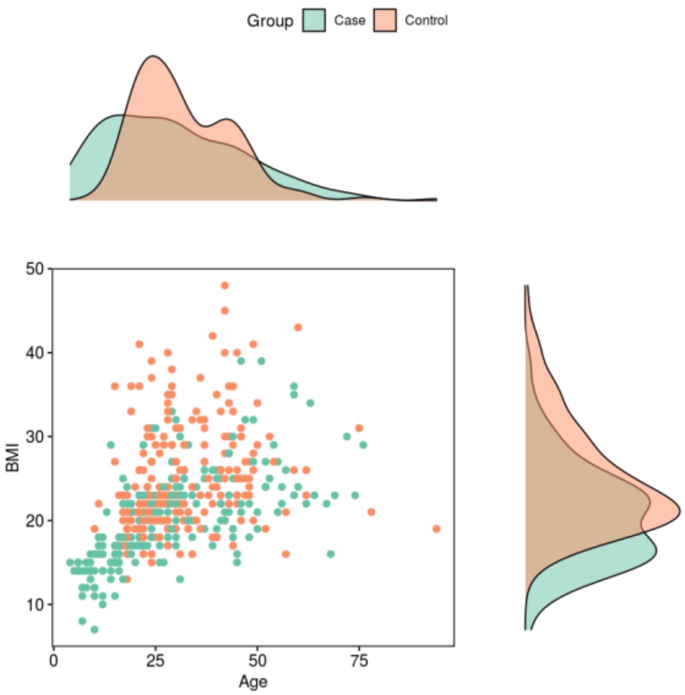

Supplementary Fig. 1 Scatter plots of BMI and age in cases and controls with their density plots in each group.

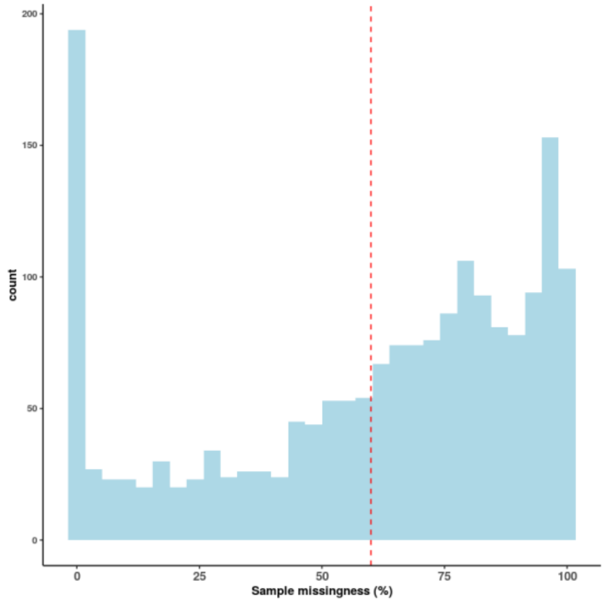

Supplementary Fig. 2 Percentage of missing data in proteins with dashed red line representing the cutoff line.

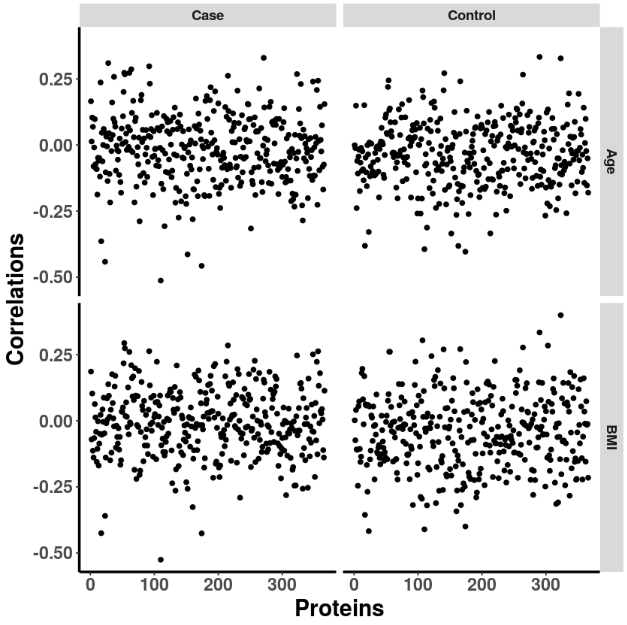

Supplementary Fig. 3 Pearson correlation coefficients of protein expression with BMI/Age among case and control samples. Correlation coefficients are in general weak, and not systematically different between cases and controls.

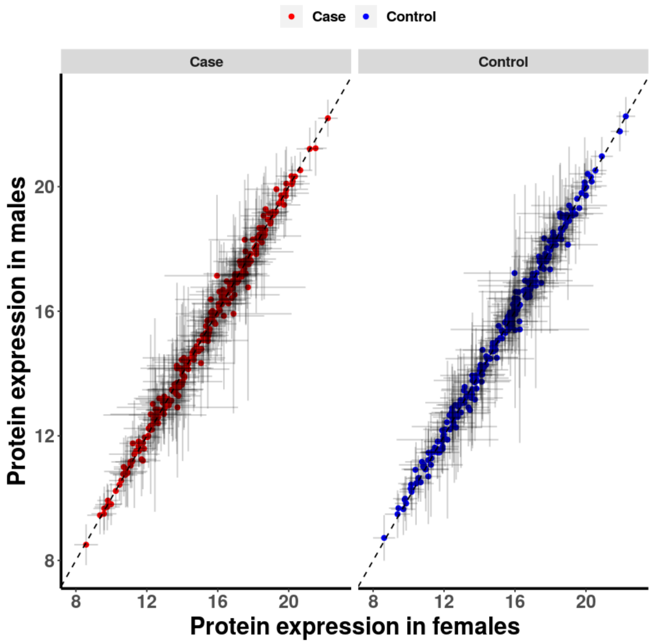

Supplementary Fig. 4 Errorbar plots of protein expressions in females (x-axis) and males (y-axis) in case and control samples, respectively. The dashed lines are identity lines.

Supplementary Table 1. The eight African countries contributing participants in the study

Country	No. of cases	No. of controls	Total
Kenya	48	28	76
Mozambique	20	6	26
Namibia	23	24	47
Nigeria	13	4	17
South Africa	41	100	141
Sudan	11	20	31
Uganda	35	24	59
Zambia	24	24	48
**Total**	**215**	**230**	**445**

Supplementary Table 2 Mean of log2-scaled protein expression quantities in cases and controls, log2 fold-change between cases and controls, p-values from t-test and adjusted p-values for multiple comparisons (only proteins with adjusted p value<0.05 are shown here).

Protein	Protein Name	Mean of log2-scaled expression in cases	Mean of log2-scaled expression in controls	Log2 fold-change	p_value	adj_p_value
P02741	CRP	13.9	12.77	1.17	4.49e-08	1.76e-05
P0DJI8	SAA1	12.3	11.20	1.14	7.99e-06	3.13e-03
Q15848	ADIPOQ	11.2	10.08	1.14	6.49e-22	2.55e-19
P20742	PZP	16.6	15.79	0.85	2.84e-16	1.11e-13
P07237	P4HB	16.4	15.69	0.72	1.24e-10	4.87e-08
P10643	C7	16.7	16.00	0.72	4.47e-22	1.75e-19
O75369	FLNB	18.9	18.23	0.72	1.82e-09	7.14e-07
P00338	LDHA	16.7	16.10	0.63	9.65e-06	3.78e-03
P04424	ASL	16.3	15.70	0.60	6.73e-07	2.64e-04
P42330	AKR1C3	14.7	14.08	0.60	6.55e-05	2.57e-02
P50225	SULT1A1	11.6	10.98	0.59	1.92e-06	7.52e-04
P19320	VCAM1	10.8	10.19	0.58	1.63e-07	6.38e-05
P04211	IGLV7-43	13.1	12.50	0.56	9.26e-05	3.63e-02
P23142	FBLN1	13.9	13.31	0.55	3.75e-13	1.47e-10
Q8NBP7	PCSK9	14.1	13.53	0.53	2.30e-11	9.00e-09
O00391	QSOX1	12.5	11.96	0.52	2.49e-19	9.77e-17
P22307	SCP2	14.6	14.09	0.50	2.10e-08	8.24e-06
P07333	CSF1R	11.1	10.59	0.49	1.35e-12	5.29e-10
P61769	B2M	12.0	11.53	0.48	5.85e-13	2.29e-10
P11413	G6PD	18.3	17.83	0.47	1.82e-11	7.15e-09
Q99714	HSD17B10	11.9	11.48	0.47	8.83e-08	3.46e-05
P61626	LYZ	12.5	12.00	0.46	1.92e-09	7.54e-07
P31943	HNRNPH1	16.2	15.75	0.44	1.22e-05	4.78e-03
P00450	CP	20.3	19.91	0.44	4.50e-11	1.76e-08
O14980	XPO1	15.7	15.31	0.43	7.39e-06	2.90e-03
Q13564	NAE1	16.2	15.76	0.42	7.11e-06	2.79e-03
Q9H4G4	GLIPR2	12.7	12.28	0.42	4.12e-11	1.61e-08
P35442	THBS2	12.2	11.83	0.42	2.16e-06	8.47e-04
Q5JRA6	MIA3	10.2	9.83	0.41	1.65e-05	6.45e-03
Q99784	OLFM1	10.9	10.53	0.40	7.67e-11	3.01e-08
O43707	ACTN4	18.5	18.14	0.40	6.26e-08	2.46e-05
P02750	LRG1	17.1	16.72	0.39	4.69e-06	1.84e-03
P30041	PRDX6	14.8	14.44	0.37	2.16e-10	8.46e-08

P31939	ATIC	16.6	16.26	0.37	1.96e-06	7.68e-04
Q06033	ITIH3	15.5	15.16	0.35	1.95e-07	7.65e-05
Q14766	LTBP1	12.1	11.73	0.35	6.73e-05	2.64e-02
Q86VB7	CD163	11.0	10.66	0.34	8.85e-07	3.47e-04
Q8NBS9	TXNDC5	15.9	15.59	0.34	8.67e-05	3.40e-02
P27824	CANX	15.8	15.49	0.33	3.64e-05	1.43e-02
P02792	FTL	14.6	14.25	0.32	5.06e-07	1.98e-04
Q12805	EFEMP1	12.4	12.12	0.30	2.39e-07	9.36e-05
P01011	SERPINA3	21.2	20.92	0.27	3.02e-05	1.18e-02
P43251	BTD	14.2	14.43	-0.20	2.04e-05	8.01e-03
Q9UK55	SERPINA10	12.9	13.13	-0.22	1.18e-05	4.63e-03
P04180	LCAT	13.0	13.24	-0.22	5.98e-06	2.35e-03
Q6EMK4	VASN	11.1	11.37	-0.23	4.43e-06	1.74e-03
Q13790	APOF	11.7	11.97	-0.23	3.31e-05	1.30e-02
P10909	CLU	17.8	18.09	-0.25	7.22e-05	2.83e-02
P00747	PLG	18.6	18.85	-0.25	1.25e-04	4.89e-02
P27918	CFP	12.3	12.52	-0.25	3.32e-05	1.30e-02
P04004	VTN	19.0	19.22	-0.27	3.43e-05	1.34e-02
P49908	SELENOP	11.7	11.95	-0.27	1.93e-06	7.58e-04
P43652	AFM	18.4	18.65	-0.28	5.10e-05	2.00e-02
Q92954	PRG4	11.2	11.45	-0.28	8.13e-06	3.19e-03
P19823	ITIH2	19.3	19.54	-0.28	1.06e-05	4.17e-03
P05154	SERPINA5	12.2	12.51	-0.31	7.47e-06	2.93e-03
P19827	ITIH1	18.7	19.01	-0.34	2.43e-08	9.52e-06
P03952	KLKB1	15.7	16.10	-0.35	8.18e-09	3.21e-06
P17936	IGFBP3	13.8	14.11	-0.36	6.23e-08	2.44e-05
P02753	RBP4	17.2	17.58	-0.37	6.56e-06	2.57e-03
P05452	CLEC3B	14.4	14.77	-0.37	7.27e-09	2.85e-06
Q6UX04	CWC27	14.4	14.74	-0.38	1.11e-06	4.37e-04
P29622	SERPINA4	15.2	15.55	-0.39	1.11e-09	4.36e-07
P62701	RPS4X	12.9	13.36	-0.42	4.40e-13	1.73e-10
O14791	APOL1	13.3	13.73	-0.42	1.28e-08	5.01e-06
P02790	HPX	21.4	21.87	-0.44	6.27e-12	2.46e-09
P02749	APOH	18.5	18.98	-0.44	1.27e-11	4.96e-09
P05546	SERPIND1	18.1	18.52	-0.45	5.52e-10	2.16e-07
P02655	APOC2	15.8	16.24	-0.45	1.75e-05	6.85e-03
O95445	APOM	16.1	16.54	-0.45	4.80e-09	1.88e-06
P36955	SERPINF1	15.2	15.66	-0.47	3.48e-15	1.36e-12
P06276	BCHE	13.6	14.07	-0.48	1.64e-12	6.44e-10
P05090	APOD	17.0	17.48	-0.49	7.84e-10	3.07e-07
P25311	AZGP1	16.6	17.07	-0.50	2.40e-13	9.41e-11
P02654	APOC1	15.5	15.95	-0.50	5.58e-08	2.19e-05
P27169	PON1	16.1	16.63	-0.55	1.41e-12	5.54e-10
Q9Y6U3	SCIN	15.3	15.83	-0.57	5.33e-15	2.09e-12
P06396	GSN	16.9	17.49	-0.57	1.57e-14	6.15e-12
P35858	IGFALS	15.4	15.99	-0.60	1.94e-16	7.60e-14
P02766	TTR	17.6	18.21	-0.60	2.73e-11	1.07e-08
P02743	APCS	16.3	16.92	-0.60	1.00e-16	3.93e-14
P02656	APOC3	15.3	15.93	-0.61	1.42e-07	5.57e-05
P80108	GPLD1	12.6	13.30	-0.67	7.38e-17	2.89e-14
O75636	FCN3	14.0	14.82	-0.81	1.03e-19	4.05e-17

Supplementary Table 3 Comparisons of protein signatures identified by Boruta algorithm and LASSO regression

UniProtID	ProteinName	meanImp	label
Q15848	ADIPOQ	13.67	Lasso&Boruta
P10643	C7	11.76	Lasso&Boruta
O00391	QSOX1	9.95	Boruta
P35858	IGFALS	9.2	Lasso&Boruta
P20742	PZP	8.95	Lasso&Boruta
P80108	GPLD1	8.45	Lasso&Boruta
P23142	FBLN1	7.49	Lasso&Boruta
P25311	AZGP1	7	Lasso&Boruta
P36955	SERPINF1	6.66	Boruta
P06396	GSN	6.63	Lasso&Boruta
P00450	CP	6.39	Lasso&Boruta
Q99784	OLFM1	6.06	Lasso&Boruta
P02743	APCS	6.04	Lasso&Boruta
P19320	VCAM1	5.95	Boruta
P02749	APOH	5.94	Boruta
P61626	LYZ	5.78	Lasso&Boruta
O75636	FCN3	5.6	Lasso&Boruta
P30041	PRDX6	5.44	Lasso&Boruta
P05546	SERPIND1	5.31	Boruta
P07333	CSF1R	5.26	Boruta
P51884	LUM	5.15	Lasso&Boruta
Q06033	ITIH3	5.08	Boruta
P07237	P4HB	5.08	Boruta
P05090	APOD	4.9	Lasso&Boruta
P02766	TTR	4.73	Boruta
P62701	RPS4X	4.61	Boruta
P02741	CRP	4.27	Lasso&Boruta
P11413	G6PD	4.25	Boruta
P61769	B2M	4.25	Lasso&Boruta
Q9UK55	SERPINA10	4.13	Boruta
P29622	SERPINA4	4.08	Boruta
P02790	HPX	4.07	Boruta
Q86VB7	CD163	3.97	Boruta
O95445	APOM	3.92	Boruta
P17948	FLT1	3.91	Boruta
Q9Y6U3	SCIN	3.8	Lasso&Boruta
O75369	FLNB	3.64	Lasso&Boruta
P35442	THBS2	3.63	Lasso&Boruta
P02750	LRG1	3.39	Boruta
O14791	APOL1	3.36	Lasso&Boruta
P06276	BCHE	3.35	Boruta
P04424	ASL	3.31	Boruta
P02654	APOC1	3.26	Boruta
P05186	ALPL	3.25	Boruta
O43707	ACTN4	3.24	Lasso&Boruta
P27169	PON1	3.22	Lasso&Boruta
P19827	ITIH1	3.16	Boruta
P32119	PRDX2	3.14	Boruta
O14980	XPO1	3.09	Boruta
P03952	KLKB1	3.07	Boruta

Q6UX04	CWC27	3.04	Boruta
P02656	APOC3	2.96	Boruta
Q9H4G4	GLIPR2	2.93	Boruta
P19823	ITIH2	2.92	Boruta
P22307	SCP2	2.89	Boruta
P17936	IGFBP3	2.88	Boruta
Q8IZ83	ALDH16A1	2.43	Lasso
P62158	CALM1	2.09	Lasso
P62158	CALM2	2.09	Lasso
P62158	CALM3	2.09	Lasso
Q9HC38	GLOD4	1.95	Lasso
P05452	CLEC3B	1.49	Lasso
Q99714	HSD17B10	1.26	Lasso
P30084	ECHS1	1.02	Lasso
P29350	PTPN6	1	Lasso
Q5JRA6	MIA3	0.97	Lasso
O75390	CS	0.85	Lasso
P04275	VWF	0.76	Lasso
Q9NTJ3	SMC4	0.75	Lasso
P52566	ARHGDIB	0.62	Lasso
P07737	PFN1	0.41	Lasso
P15144	ANPEP	0.39	Lasso
Q09666	AHNAK	0.22	Lasso
P02042	HBD	0.1	Lasso
Q13576	IQGAP2	0.03	Lasso
P01871	IGHM	-0.02	Lasso
Q9Y4L1	HYOU1	-0.13	Lasso
P61764	STXBP1	-0.15	Lasso
P12277	CKB	-0.17	Lasso
P39019	RPS19	-0.35	Lasso
Q02790	FKBP4	-0.56	Lasso

Supplementary Table 4 Pathways from ClueGO analysis of input proteins identified by Bortua algorithm by using plasma library reference (n=2,559)

REACTOME Pathways.

**Term**	**P value**	**P_FDR_adj**	**#Genes**
**Regulation of Insulin-like Growth Factor (IGF) transport and uptake by Insulin-like Growth Factor Binding Proteins (IGFBPs)**	**4.16e-05**	**0.00017**	**9**
**Post-translational protein phosphorylation**	**4.16e-05**	**0.00017**	**8**
**Amyloid fiber formation**	**0.00011**	**0.00011**	**5**

GO Biological Processes

**Term**	**P value**	**P_FDR_adj**	**#Genes**
**serine-type endopeptidase inhibitor activity**	**9.89e-06**	**4.94e-05**	**8**

**negative regulation of cell population proliferation**	**6.62e-07**	**3.97e-06**	**14**
**modulation by symbiont of entry into host**	**7.88e-05**	**0.00016**	**4**
**regulation of viral entry into host cell**	**7.88e-05**	**0.00016**	**4**
**regulation of plasma lipoprotein particle levels**	**4.44e-05**	**0.00013**	**6**
**lipid metabolic process**	**4.44e-05**	**0.00013**	**16**
**lipid localization**	**4.44e-05**	**0.00013**	**10**
**lipid transport**	**4.44e-05**	**0.00013**	**9**
**lipoprotein metabolic process**	**4.44e-05**	**0.00013**	**6**
**negative regulation of lipid metabolic process**	**4.44e-05**	**0.00013**	**4**
**plasma lipoprotein particle assembly**	**4.44e-05**	**0.00013**	**4**
**sterol transport**	**4.44e-05**	**0.00013**	**6**
**negative regulation of lipid catabolic process**	**4.44e-05**	**0.00013**	**3**
**very-low-density lipoprotein particle assembly**	**4.44e-05**	**0.00013**	**3**
**regulation of sterol transport**	**4.44e-05**	**0.00013**	**5**
**cholesterol transport**	**4.44e-05**	**0.00013**	**6**
**regulation of cholesterol transport**	**4.44e-05**	**0.00013**	**5**
**cholesterol efflux**	**4.44e-05**	**0.00013**	**5**

Supplementary Table 5 Pathways from ClueGO analysis of input proteins identified by Bortua algorithm by using whole human genome reference.

KEGG Pathways

**Term**	**P value**	**P_FDR_adj**	**# Genes**
Cholesterol metabolism	0.00039	0.0046	6

REACTOME Pathways

**Term**	**P value**	**P_FDR_adj**	**#Genes**
Complement cascade	0.00060	0.0048	3
Plasma lipoprotein assembly, remodeling, and clearance	0.0010	0.0020	3
Scavenging of heme from plasma	6.08e-06	0.00024	3
Binding and Uptake of Ligands by Scavenger Receptors	0.00023	0.0041	3
Detoxification of Reactive Oxygen Species	0.00016	0.00299	3
Regulation of Insulin-like Growth Factor (IGF) transport and uptake by Insulin-like Growth Factor Binding Proteins (IGFBPs)	7.27e-11	3.93e-09	9
Metabolism of fat-soluble vitamins	0.00032	0.0045	3
Post-translational protein phosphorylation	7.10e-10	3.69e-08	8
Plasma lipoprotein assembly	1.72e-05	0.00055	3
Retinoid metabolism and transport	0.00025	0.0042	3
Amyloid fiber formation	1.44e-05	0.00049	5

WIKI Pathways

Term	P value	P_FDR_adj	#Genes
Human Complement System	8.98e-06	0.00033	5
PPAR signaling pathway	0.00091	0.0037	3
Complement and Coagulation Cascades	0.00063	0.0044	3

GO Biological Processes

Term	P value	P_FDR_adj	#Genes
serine-type endopeptidase inhibitor activity	4.85e-10	2.57e-08	8
endopeptidase inhibitor activity	8.10e-08	4.13e-06	8
cholesterol transport	2.67e-07	1.34e-05	6
cholesterol efflux	2.96e-07	1.45e-05	5
regulation of plasma lipoprotein particle levels	3.22e-07	1.55e-05	6
regulation of cholesterol transport	5.26e-07	2.47e-05	5
sterol transport	5.77e-07	2.60e-05	6
regulation of sterol transport	5.75e-07	2.65e-05	5
plasma lipoprotein particle clearance	6.85e-07	3.01e-05	5
plasma lipoprotein particle assembly	2.54e-06	0.00011	4
regulation of viral entry into host cell	3.59e-06	0.00015	4
lipoprotein metabolic process	3.56e-06	0.00015	6
protein-lipid complex assembly	4.01e-06	0.00016	4
modulation by symbiont of entry into host	7.27e-06	0.00028	4
very-low-density lipoprotein particle assembly	9.63e-06	0.00035	3
high-density lipoprotein particle clearance	1.18e-05	0.00041	3
plasma lipoprotein particle organization	1.66e-05	0.00055	4
high-density lipoprotein particle remodeling	1.72e-05	0.00055	3
regulation of lipoprotein particle clearance	2.03e-05	0.00063	3
negative regulation of viral entry into host cell	2.03e-05	0.00063	3
protein-lipid complex subunit organization	2.21e-05	0.00066	4
regulation of lipid catabolic process	3.47e-05	0.0010	4
modulation by host of symbiont process	0.0011	0.0011	3
regulation of lipoprotein lipase activity	4.21e-05	0.0012	3
glycerolipid catabolic process	6.43e-05	0.0017	4
negative regulation of lipid catabolic process	6.76e-05	0.0017	3
positive regulation of sterol transport	8.34e-05	0.0020	3
positive regulation of cholesterol transport	8.34e-05	0.0020	3
cell redox homeostasis	9.50e-05	0.0022	4
protein-lipid complex remodeling	0.00010	0.0022	3
plasma lipoprotein particle remodeling	0.00010	0.0022	3
protein activation cascade	0.00010	0.0022	3
blood coagulation, fibrin clot formation	0.00010	0.0022	3
protein-containing complex remodeling	0.00011	0.0023	3
negative regulation of cell-substrate adhesion	0.00095	0.0029	3

modulation by host of viral process	0.00014	0.0029	3
hyaluronan metabolic process	0.00026	0.0042	3
regulation of fatty acid biosynthetic process	0.00043	0.0043	3
acute-phase response	0.00073	0.0044	3
regulation of substrate adhesion-dependent cell spreading	0.00063	0.0044	3
negative regulation of lipid localization	0.00034	0.0045	3
negative regulation of smooth muscle cell proliferation	0.00041	0.0045	3

GO Molecular Functions

Term	P value	P_FDR_adj	#Genes
lipoprotein lipase activity	4.77e-05	0.0013	3
triglyceride lipase activity	0.00028	0.0042	3
cholesterol binding	0.00049	0.0044	3
sterol binding	0.00076	0.0038	3

List of RHDGen Network Consortium Members

THE GENETICS OF RHEUMATIC HEART DISEASE (RHDGEN) NETWORK CONSORTIUM

Founding Senior PI: Bongani M Mayosi, MD DPhil

Study conceptualization/design team (RHDGen grant: 099313/B/12/A): B M Mayosi, H J Cordell, J de Vries, A El Sayed, ME Engel, CT Hugo-Hamman, M Lesosky, B Keavney, A Mocumbi, C Mondo, N Mulder, J Musuku, G Paré, Raj Ramesar, O Ogah, S Ogendo, G Shaboodien;

INVESTIGATORS, COMMITTEES, AND STAFF

Investigators – number of patients enrolled in each country and site are in parenthesis

South Africa (2473): The Cardiac Clinic, Groote Schuur Hospital, Cape Town (2216) – M Ntsekhe, C Chishala, B Cupido; Livingstone and Provincial Hospitals, Port Elizabeth (257) - L Pepeta; *Namibia (743):* Rheumatic Heart Disease Clinic, Windhoek Central Hospital, Ministry of Health and Social Services, Windhoek, Republic of Namibia – CT Hugo-Hamman, T Auala, C Brinkman, L Sikwaya; *Kenya (642)* - Cardiology Department of Medicine, Kenyatta National Hospital, University of Nairobi – Bernard M Gitura, Stephen Ogendo, Tom Omulo; *Uganda (568):* Uganda Heart Institute, Departments of Adult and Peadiatric cardiology, Kampala, Uganda – P Lwabi, E Okello; *Mozambique (557):* Faculty of Medicine, Eduardo Mondlane University / Nucleo de Investigaçao,

Departamento de Medicina, Hospital Central de Maputo, Maputo, Mozambique (367) - A Damasceno, A Fabula; Instituto Nacional de Saúde Ministério da Saúde – Moçambique (190) – A Mocumbi, G Madeira; *Zambia (548):* University Teaching Hospital – Children's Hospital, University of Zambia, Lusaka, Zambia - J Musuku, A Mtaja; *Sudan (488):* Department of Cardiothoracic Surgery, Alshaab Teaching Hospital, Alazhari Health Research Center, Alzaiem Alazhari University, Khartoum, Sudan – A ElSayed, HHM Alhassan, A Abdelhalim, H Alhajapo, E Idris, S Salih; *Nigeria (335):* Departments of Paediatrics and Medicine, Jos University Teaching Hospital and University of Jos, Jos, Plateau State Nigeria – F Bode-Thomas, B Okeahialam, S Danbauchi, O Ige, C Yilgwan, G Amusa, E Nkereuwen.

Project Coordinating Office, University of Cape Town, South Africa

ME Engel (Study Manager), R Daniels (Research Assistant), J de Vries (Ethicist), V Francis (Coordinator), F. Gili (Research Assistant), P Kraba (Research Assistant), S Pandie (Data Manager), R. Vergotine (Data Assistant);

Project Coordinating Laboratory, Cardiovascular Genetics, Hatter Institutefor Cardiovascular Research in Africa & Cape Heart Institute (CHI), University of Cape Town, South Africa

G Shaboodien (Director), S Kamuli (Laboratory Scientist), T Machipisa (Laboratory Scientist), B Muhamed (Laboratory Scientist), L Pearce (Research Assistant), J Saaiman (Research Assistant);

Bioethics Research Initiative, Department of Medicine, University of Cape Town, South Africa

J de Vries (Director), Research Fellows: M Faure, F Masiye, O P Matshabane, N S Munung;

Population Health Research Institute (PHRI), Hamilton Health Sciences and McMaster University RHDGen Project Team (Genetic Molecular Epidemiology Laboratory, GMEL)

G Paré (GMEL Director), Michael Chong (Bioinformatician), R Ditta (Laboratory Coordinator), A Hodge (Laboratory Technician), T Machipisa (visiting RHDGen Fellow);

Research Assistants, Coordinators, Fieldworkers

South Africa: The Cardiac Clinic, Groote Schuur Hospital, Cape Town – U September (Research Nurse), S Nkepu (Field Site Coordinator), M Van de Wall (Echocardiographer), N Laing (Genetic Counsellor), A Joachim (Research Nurse); Livingstone and Provincial Hospitals, Port Elizabeth (257) – R Solwandle (Research Nurse); *Namibia (743):* Rheumatic Heart Disease Clinic, Windhoek Central Hospital, Ministry of Health and Social Services, Windhoek, Republic of Namibia – G Olivier (Field Site Administrator), A Awases (Research Nurse), A Bock (Research Nurse), S Nzuza (Clinical Technologist), C Mangami (Clinical Technologist), H Amanyanga (Research Nurse), A Mneumbo (Research Nurse); *Kenya (642) -* Cardiology Department of Medicine, Kenyatta National Hospital, University of Nairobi – Elizabeth Musyoki (Research Nurse); *Uganda (568): Uganda (568):* Uganda Heart Institute, Departments of Adult and Peadiatric cardiology, Kampala, Uganda – I Ssinabulya (Clinician Researcher), S Kitoleko (Research Nurse), J Kebba

(Labarotory Technologist); *Mozambique (557):* Faculty of Medicine, Eduardo Mondlane University / Nucleo de Investigaçao, Departamento de Medicina, Hospital Central de Maputo, Maputo, Mozambique (367) - C Novela (Research Coordinator), M Machava (Research Assistant), V Govo (Research Assistant); Instituto Nacional de Saúde Ministério da Saúde – Moçambique (190) – S Candido (Research Nurse); *Zambia (548):* University Teaching Hospital – Children's Hospital, University of Zambia, Lusaka, Zambia; A Lungu (Research Nurse), T Kaira (Laboratory Technologist); *Sudan (488):* Department of Cardiothoracic Surgery, Al Shaab Teaching Hospital, Faculty of Medicine, Alzaiem Alazhari University, Khartoum, Sudan – NKM Elkhder (Radiographer); *Nigeria (335):* Departments of Paediatrics and Medicine, Jos University Teaching Hospital and University of Jos, Jos, Plateau State Nigeria – C Barau (Research Nurse), OA Marcaulay (Research Assistant), D Badung (Laboratory Scientist), C Durojaiye-Amodu (Data Clerk).
